# Etiology and Measurement of Peri-Implant Crestal Bone Loss (CBL)

**DOI:** 10.3390/jcm8020166

**Published:** 2019-02-01

**Authors:** Adrien Naveau, Kouhei Shinmyouzu, Colman Moore, Limor Avivi-Arber, Jesse Jokerst, Sreenivas Koka

**Affiliations:** 1 Department of Prosthodontics, Dental Science Faculty, University of Bordeaux, 33000 Bordeaux, France; Adrien.naveau@laposte.net; 2 Dental and Periodontal Rehabilitation Unit, Saint Andre Hospital, Bordeaux University Hospital, 33000 Bordeaux, France; 3 Department of Oral Implants, Kyushu Dental University, Kitakyushu, Fukuoka 803-8580, Japan; k.shinmyouzu@spice.ocn.ne.jp; 4 Tanpopo Dental Clinic, Nerima ward, Tokyo 178-0062, Japan; 5 Department of NanoEngineering, University of California San Diego, La Jolla, CA 92093, USA; cam081@eng.ucsd.edu (C.M.); jjokerst@eng.ucsd.edu (J.J.); 6 Faculty of Dentistry, University of Toronto, Toronto M5G1G6, ON M5G 1G6, Canada; Limor.Avivi-Arber@dentistry.utoronto.ca; 7 Materials Science Program, University of California San Diego, La Jolla, CA 92093, USA; 8 Department of Radiology, University of California San Diego, La Jolla, CA 92093, USA; 9 Private practice, Koka Dental Clinic, San Diego, CA 92111, USA; 10 Advanced Prosthodontics, Loma Linda University School of Dentistry, Loma Linda, CA 92350, USA; 11 Advanced Prosthodontics, University of California Los Angeles School of Dentistry, Los Angeles, CA 90095, USA

**Keywords:** Crestal bone loss, osseosufficiency, osseoseparation, peri-implantitis, photoacoustic ultrasound, brain–bone axis, foreign body reaction, overloading, radiography, CBCT (cone beam computerized tomography)

## Abstract

The etiology of peri-implant crestal bone loss is today better understood and certain factors proposed in the past have turned out to not be of concern. Regardless, the incidence of crestal bone loss remains higher than necessary and this paper reviews current theory on the etiology with a special emphasis on traditional and innovative methods to assess the level of crestal bone around dental implants that will enable greater sensitivity and specificity and significantly reduce variability in bone loss measurement.

## 1. Introduction

Crestal bone loss (CBL) was relatively uncommon and non-progressing with the commercially pure titanium implants with a machined surface introduced by Per-Ingvar Branemark. It was accepted in the late 1980s and 1990s that 1 mm of CBL could be expected in the first year after implant placement and then 0.2 mm of CBL on average might occur after that. In fact, an adage took hold that with these implants, CBL to between the first and second thread is common after which time bone levels remained remarkably stable for years.

Predictably, as the application of the initial wave of implants was so successful, an expansion of clinical scenarios amenable to dental implant therapy took place. Following on, an expansion of the clinical provider pool considered appropriate to place and restore implants took place. Finally, “innovations” to the dental implant systems with the goal of fostering the expansion of clinical scenarios and provider pool also took place. Unfortunately, despite the noblest of intentions, and indeed some not so noble, the number of complications associated with dental implant therapy reported today is high. Indeed, it is far higher than necessary, and puts patients at unnecessary, and hence unjustifiable, risk for suboptimal clinical outcomes including implant loss, biological tissue loss, financial loss, and psychological trauma.

Koka and Zarb first proposed the concept of osseosufficiency to describe the role of the interplay between clinician, patient, and implant system inasmuch as promoting and perpetuating osseointegration [1]. In this model, if the combination of the ingredients that clinician (skill, knowledge, experience), patient (genetic, environmental, behavioral), and implant system (design, material) are “enough” to promote and perpetuate osseointegration, a state of osseousufficiency is attained. If the combination of ingredients is “not enough”, a state of osseoinsufficiency results. Although it is commonplace to attribute implant loss as representing “implant failure”, this is clearly not the case in most instances where implants are retrieved from jawbones, the main exception being when an implant body fractures. To state that an implant failed implies that the implant was at fault for its retrieval and assigns blame for the undesirable outcome to the one ingredient in the osseointegration recipe that is the most predictable and by far, compared to patient and clinician, the least variable. Conveniently, it is also the one element that is unable to defend itself in conversations about why an implant was retrieved. Clearly, albeit an uncomfortable state of affairs, most complications in implant therapy are clinician-dependent (inexperience, incompetence, or ignorance) and the remainder are patient-dependent or a combination of clinician and patient factors. Therefore, throughout this manuscript, the term “implant failure” will not be used. In the place of “implant failure”, terms like “implant retrieval”, “implant removal”, or “implant loss” will be used to more accurately describe the clinical outcome and to avoid inaccurate assignment of blame.

One manifestation of osseoinsufficiency that has significant clinical ramifications is peri-implant CBL because it can lead to implant retrieval, osseous deformation, soft tissue deformation, esthetic compromise, and a dissatisfied/upset patient who loses confidence in their clinical provider. Clearly, prevention is better than cure when it comes to peri-implant CBL as effective and predictable methods to restore lost bone remain elusive.

Crestal bone loss has been postulated to have a multi-factorial etiology [2] and can be considered to occur early or late in the lifetime of a dental implant. Here, early means within the first year after placement and CBL observed is a consequence of bone remodeling subsequent to surgical and restorative procedures and early loading challenges undertaken by an implant and its associated prosthesis [2,3]. Given the role of adaptive bone remodeling, early CBL is not necessarily influenced by infection from oral microflora. Over the longer term, the cumulative effect of chronic etiological factors that are immunological (foreign body reaction), environmental, including patient factors such as motivation, smoking, bruxism, and infection/inflammation, and the influence of clinician (surgeon/prosthodontist) may influence late CBL [2,3,4,5]. Given that other manuscripts in this volume will address different etiological factors of CBL, in Section A, this manuscript will provide a summary of current knowledge related to two common etiological factors, mechanical overloading and periodontopathogens/perimplantopathogens/bacteria. It will also discuss the role of the immune system through the foreign body reaction mechanisms that lie at the heart of osseointegration, and describe how adverse immune reactions and a tantalizing new potential mechanism involving the brain–bone axis may lead to CBL. In Section B, it will focus on a key and related issue of how CBL is currently measured and how it can be improved in the future. 

## 2. Section A. Selected Etiological Factors in Crestal Bone Loss

### 2.1. Overloading

After an implant body osseointegrates and is exposed to functional loading, Esposito et al. reported that overload of the implant prosthesis may lead to implant loss. Furthermore, the report suggests that overload contributes to peri-implantitis and is one of the major determinants of late implant retrieval [6].

There are a wide variety of experimental reports about overloading and implant therapy including computer simulations, such as finite element analysis, and in vivo and in vitro experiment. However, the results are inconclusive regarding the strength and validity of the evidence clearly linking overloading to CBL. Here, we consider the clinical significance of overloading in peri-implant CBL.

What is ‘overloading’? ‘Overloading’ is difficult to describe but could be considered to be the force level and/or nature of force application that exceeds the permissible or tolerable range of the prosthetic and biological resistance to CBL. Each patient presents with a unique prosthetic and biological resistance profile and reference ranges of permissibility are, as yet, unknown. Hence, predicting who will be more or less susceptible to the effects of overloading is difficult. Most reports draw a conclusion of overloading based on the findings of a complication (fracture of the prosthesis, marginal bone loss etc.) as a post hoc event. Nevertheless, the complication is a result of distortion between implant and marginal bone interface caused by stress applied to the structural components of the implant prosthesis instead of the occlusal bite force itself. 

**Figure 1:**
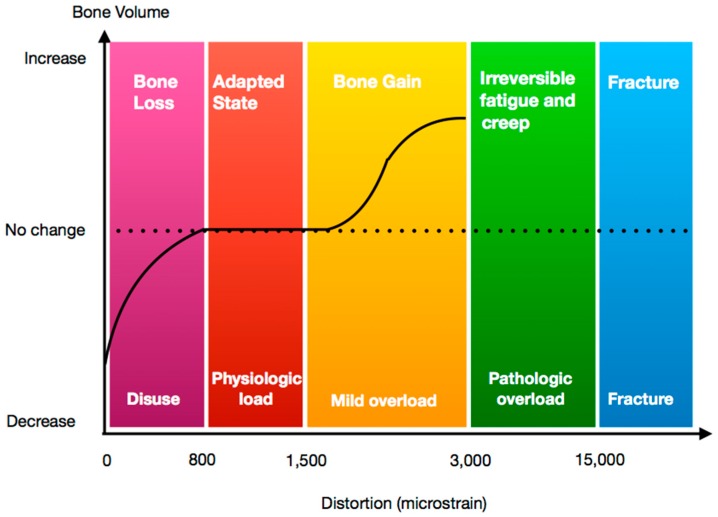
Diagram showing clinical effect on bone relative to strain level applied.

Notationally, 0.1% deformation in volume is transcribed to 1000 με (microstrain). Frost et al. divided the reaction of bone as a result of strain application into four phases or “windows” according to the amount of distortion between bone and implant (Figure 1). (i) Disuse atrophy window (50–100 με). Bone resorption may result in this phase where the net effect of bone formation and resorption is negative; (ii) Steady state window (100–1500 με). Here, the net volume of the bone remains steady; (iii) Mild overload window (1500–3000 με). Here, the net effect of bone formation and resorption is positive and bone volume increases; (iv) Fatigue failure window (>3000 με). Here, bone resorption and destruction occur [6]. 

Theoretically, when we classify the reaction of bone to force application/distortion at the implant–bone interface, we can use Frost’s definition of overload, the fatigue failure window. However, Naert et al. noted that the definition of overload in implant dentistry is more complex, open to interpretation and they suggested the range of distortion represented by Frost’s fatigue failure window does not accurately represent the over load in the clinical situation [7].

Past reports focused on overload and CBL are presented in Table 1. Isidor et al. reported on crestal bone reaction following excessive occlusal load or plaque accumulation in monkeys. In this report, 6 months after insertion of implants, a fixed partial prosthesis was mounted and there were two experimental groups: Excessive occlusal over load and plaque accumulation. A loss of osseointegration and/or CBL was observed 4.5 months to 15.5 months after overloading was initiated. None of the implants with plaque accumulation experienced CBL [8]. Miyata et al. reported the influence of controlled occlusal overload on peri-implant tissue, again in monkeys and in this model, supra-occlusal contact was applied for four weeks to implants starting fourteen weeks after insertion. Neither inflammation nor CBL was observed when supra-occlusal contact was of approximately 100 microns. In contrast, CBL was observed in the group with supra-occlusal contact was over 180 microns. The authors concluded that peri-implant CBL occurred with 180 microns or more of supra-occlusion [9]. Esaki et al. reported the relationship between the magnitude of immediate loading and peri-implant osteogenesis in a canine model [10]. In this report, immediate load (0 N, 10 N, 50 N) using a cyclic loading device was applied to implants placed in healed sites. In the 10 N group, newly formed bone was observed over a wide area from the implant neck toward the tip. In contrast, in the 50 N group, newly formed bone was rarely observed around the neck and signs of infection were seen. The authors suggested there is a certain load that is beneficial and promotes osteogenesis and an overload threshold that is detrimental. Heitz-Mayfield et al. evaluated the effect of excessive occlusal load following placement of implants in dog [11]. After six months of healing after implant insertion, supra-occlusal crowns were placed. At eight months, all implants were osseointegrated with no statistically difference between test and control implants observed with regard to osseous response.

Although each of the experiments described above employed different experimental models, taken together, it is clear that certain dynamic force applications influence CBL and bone formation around implants. Managing occlusal loading to achieve desirable effects and prevent undesirable effects is an important consideration during treatment planning and treatment.

In recent years, attention has been paid to osseous activity at the molecular level when force is applied to bone. As a result of technological advances of technology, the role of the osteocyte has become better understood.

Osteocytes are most abundant bone cell in the adult skeleton and function as mechanosensors directing osteoblast and osteoclast function in order to maintain the optimal integrity of load bearing bone. Early histologists upon observing enlarged osteocyte lacunae in bone sections proposed that mature osteocytes could remove their perilacunar matrix, a term called “osteocytic osteolysis”. New insights into this process have occurred during the last decade using novel technology thereby providing a means to identify molecular mechanisms responsible for osteocytic osteolysis [12].

Dendritic osteocytes connect to the vasculature, to each other and to periosteal bone surface cells creating a broad communication network within bone tissue. Osteocytes lie in a fluid-filled interstitium of lacunae and canaliculi and are capable of sensing when mechanical load that applied to the skeleton [13]. In response to force application, osteocytes react to and transmit information via secretion of molecules with a signaling function such as sclerostin and receptor activator of nuclear factor kappa-B ligand (RANKL) which then regulate bone matrix turnover by osteoblasts and osteoclasts [14,15]. Due to the recently discovered multi-functionality of osteocytes, ranging from phosphate homeostasis to interaction with distant organs, the regulation of the osteoblast–osteoclast axis is one mechanism by which the osteocyte network contributes to mechanosensory response to loading and may lead to osteocyte apoptosis and targeted bone resorption by osteoclasts [16]. As a result of this mechanosensing communication stream, bone resorption and targeted bone remodeling, in the absence of bacterial inflammation, may change peri-implant crestal bone contours.

### 2.2. Peri-Implant CBL and Periodontal Pathogens

The success of implant therapy ad modum Branemark in experiencing minimal CBL led to the proposal of optimistic criteria for success of implant therapy by Albrektsson et al. in 1986 [1]. These criteria were quickly accepted as clinicians and scholars worldwide were able to achieve the criteria proposed based on ≤1–2 mm of CBL in the first year after placement and ≤0.2 mm mean CBL bone loss in subsequent years. The fact that these criteria were based on clinical outcomes from implant therapy in edentulous patients/subjects was appreciated and concern remained that implants placed in partially edentulous patients with their dental reservoirs of periodontopathogens would not be able to duplicate the excellent crestal bone response seen in edentulous patients. These concerns were laid to rest by Van Steenberghe et al. who, from multi-center study findings published in 1993, clearly demonstrated that implant therapy ad modum Branemark in partially edentulous patients also exhibited the same excellent resistance to CBL as observed in edentulous patients [17].

Nevertheless, in the relatively uncommon cases of CBL seen, the concurrent peri-implant mucosal inflammation and bacterial cultures yielding traditional periodontopathogens spawned an erroneous belief system that peri-implant CBL was fashioned after the same etiology as periodontitis. This despite that nowhere else in the human body is an artificial substitute considered to be the same as the original biological tissue: A man-made substance is yet to be fabricated that is identical to natural tissue (see next section). Today, one comes across people who simultaneously claim that bone created from an allograft is different to native bone and yet who argue that periodontal bone loss is the same as peri-implant CBL and should be prevented, diagnosed, and treated similarly. The use of the term peri-implantitis has merely cemented the error of association in the minds of clinicians and scholars despite the fact that, to date, there is no clear and compelling evidence that peri-implant CBL is primarily a consequence of bacterial insult. Of course, many of the same bacteria are found in diseased periodontal and peri-implant sites, more a consequence of anaerobic environments that lend themselves to colonization and propagation of these bacteria [18]. The osseosufficiency model presents the patient as an important element of the path towards successful implant therapy. Yet, it is critical to recognize that is the host response of the patient that is important, not the presence or absence or site concentration of specific bacterial species that prevails and the host response to an artificial implant substitute is markedly different than the host response to a natural tooth. Once peri-implant CBL or inflammation is observed, addressing the bacterial component may alleviate the symptoms and signs, but it will not address the root cause of the problem which is more likely to be improper diagnosis, treatment planning and treatment by the unaware clinician that then leads to peri-implant CBL.

Further erroneous implications are engendered when research models used to study periodontitis are applied to the dental implant ecosystem, most notably, the use of ligature-induced peri-implantitis canine model that creates an artificial scenario by which bacterial inflammation is induced around implants in order to study the degree of CBL. Clinically irrelevant periods of oral hygiene cessation, sometimes 4 months in duration, are combined with the introduction of plaque-attracting sulcular ligatures in order to create a scenario that bears no resemblance to clinical practice and which, therefore, have no clinical meaning [19,20].

### 2.3. Bone and the Immune System—Foreign Body Reaction

Any foreign-body implant that is placed in contact with vital tissues can activate the immune/inflammatory systems whereby under normal conditions the defense cells, including neutrophils, lymphocytes, reactive pro-inflammatory macrophages (i.e., M1 and OsteoMac), and osteoclasts are activated and engulf and then digest the foreign body. The repair cells, such as fibroblasts, osteoblasts as well as macrophages (M2 and OsteoMacs) are also activated and assist in tissue repair and remodeling, and protection of the tissues from further destruction. However, when a foreign body is too large to be engulfed or digested by the immune cells, a fibrous (granuloma) or osseous encapsulation of the foreign body is formed around the foreign body. This encapsulation isolates the foreign body from the surrounding tissue and is characterized by a chronic presence of macrophages and multinucleated foreign body giant cells (i.e., Langerhan’s cells) at the foreign-body surface. These foreign-body giant cells are the result of fusion of monocytes and macrophages activated upon adherence to the foreign-body surface during the earlier inflammation and tissue-repair stages. While these giant cells may present throughout the foreign-body life-time, it is unclear whether they remain active or become inactive with time. Another possible immune reaction to a foreign-body occurs when the immune response is too vigorous or too prolonged, or its function is disrupted. In such situations, the defense/repair balance may shift towards chronic inflammation and chronic tissue destruction [21,22,23,24]. 

**Figure 2:**
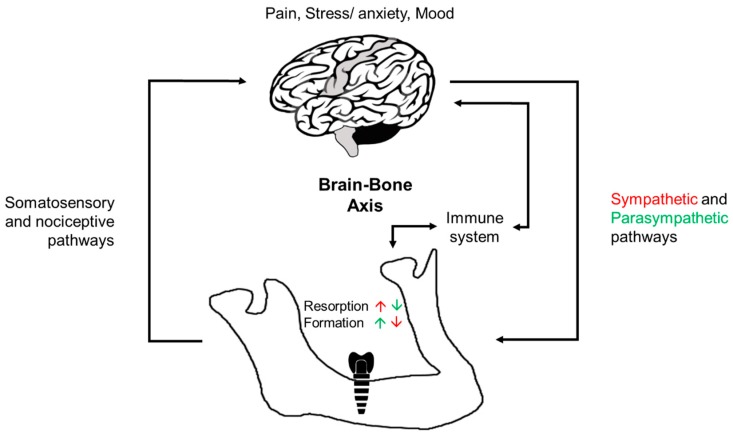
A diagram illustrating brain–bone axis involving the sympathetic and parasympathetic nervous systems that act through direct and direct neuronal innervation of bone tissue (black arrows). Centrally modulated sympathetic activity inhibits osteoblasts and bone formation and enhances osteoclast activity and bone resorption (red), while centrally modulated parasympathetic activity enhances osteoblast activity and bone formation and inhibits osteoclasts and bone resorption (green). Somatosensory and nociceptive inputs from the bone to the brain as well as pain, stress, and mood responses can impact bone formation and resorption either directly through the autonomic nervous system or indirectly through activation of the immune system that can also be activated directly by bone injury.

In the case of dental implants (see above), it is well established that when titanium implants are placed in the jaw-bone, an evoked inflammatory reaction is followed by formation of new bone in close approximation around the implant. Subsequently, a long-lasting (i.e., implant lifetime) steady state bone remodeling activity is established which maintains the bone around the implant including the marginal bone level height. This process has been named by Branemark ‘osseointegration’, whereby titanium has been considered an immunologically inert material that supports the bone healing process. It was Donath et al [25] who first suggested that in fact, this reaction of bone-tissue engulfing a dental implant is consistent with a protective foreign body immune response whereby the bone formed around the implant isolates it and thus protects the surrounding bone marrow tissue (Figure 2). This hypothesis has been further investigated and subsequently supported by the Wennerberg and Albrektsson group and others who have further suggested that once new bone is formed around the implant, maintenance of a balance between bone resorption and bone formation (i.e., ‘foreign-body equilibrium’) can maintain the osseointegration and the marginal bone height around the implants [4,21,22,26,27,28,29,30,31,32,33]. Albrektsson and colleagues have proposed a revised definition of osseointegration to state that “osseointegration is a foreign body reaction where interfacial bone is formed as a defense reaction to shield off the implant from the tissues [34] and further elucidated the importance of host response in long-term osseointegration outcomes [35]. However, the majority of the studies on foreign-body response are in vitro studies or studies that have utilized titanium or other biomaterial implants placed in limb bones or other body tissues. In vitro studies have shown that titanium can activate macrophages [30], and that complement factors in blood plasma binds to titanium implant surface which suggests that during the early stage of inflammation following titanium implant placement, the implant surfaces can be recognized by the immune cells through complement factors in the blood [36]. In the recent study in rabbits, it has been shown that the formation and subsequent maintenance of new bone around titanium implants placed in a femur bone are associated with time-dependent immune responses. These responses were manifested first (10 days) as up-regulation of immune defense cells (i.e., macrophages), and subsequently (at 28 days) by up-regulation of immune repair cells (macrophages, lymphocytes, neutrophils, and the complement system), plus down-regulation of bone-resorbing cells (osteoclasts) around the implants [33]. In addition, similar to foreign-body host response in other body parts, multinucleated giant cells are present at the dental implant–bone interface [25,37], and while these giant cells can be present throughout the implant life-time, it is unclear whether they become inactive with time, or remain active, or become active under certain conditions leading to marginal bone resorption.

Considering the intimate relations between the immune system and bone healing and remodeling, when the immune response to titanium implants is coupled with certain factors or health conditions that impact the immune system or the immune response, the balance between osteoblast and osteoclast activity can shift during the healing phase from a net bone apposition to a net bone resorption resulting in osseointegration failure or unwarranted CBL. Moreover, since osseointegration is a dynamic state of bone remodeling, these factors or health conditions may also impact the osseointegration after it had already been established. These factors or conditions include genetic factors, immunosuppressed diseases, smoking, poorly controlled surgery, excess cement, and medications [26,38,39,40,41,42]. Therefore, patient examination and medical history taking should be evaluated not only prior to implant placement, but also on a regular basis as part of the postoperative follow-up appointments. Other possible causes for impaired bone healing or marginal bone loss could be titanium ion leakage, titanium particles detachment and implant surface contamination with metal or organic particles that are residues of the implant manufacturing, implant cleaning and handling, surgical placement or prosthetic installation processes, as well as prosthetic materials [43,44]. Such particles in the bone surrounding the dental implants can induce chronic inflammatory reaction and immune response which include activation of immune cell mediators such as cytokines (e.g., tumor necrosis factor-alpha (TNF-α)) that can influence the activities of osteoblasts and osteoclasts and thereby impact on bone healing and bone turnover around the implants [29,45,46,47]. On the other hand, if osseointegration had already occurred, considering osseointegration is dynamic, the above-mentioned factors can also impact bone homeostasis and shift bone turnover into a net bone resorption manifested as aseptic osseoseparation and/or marginal bone resorption (see above). This condition is considered aseptic since its initiation does not involve the oral microbiota however, this does not rule out the possibility of a secondary bacterial infection or a bacterial-derived marginal bone resorption in individual cases [4,22,26,27,29,34,35,47,48]. 

### 2.4. The Role of the Brain in Modulating Osseointegration: Brain–Bone Axis

Novel evidence suggests that the brain and the nervous system in general play vital roles in long-bone healing and remodeling processes [49,50,51]. Complex neural networks exist between the central nervous system and the bones, and nerve fibers of sympathetic, parasympathetic, and somatic origin innervate long bones [52,53]. Furthermore, nerve-derived neuropeptides [e.g., neuropeptide Y, endocannabinoids (CB)], and neurotransmitters (e.g., norepinephrine, dopamine, serotonin, and calcitonin gene-related peptide) were found in the vicinity of long-bone cells that express receptors for these neuropeptides (e.g., β2-adrenergic, Y1 (the name of the receptor) and Y2 (the name of the receptor), CB1 and CB2) and neurotransmitters (e.g., dopamine, and serotonin). These neuropeptides, neurotransmitters and receptors can in turn contribute to the regulatory mechanisms underlying bone remodeling [49,54]. In addition, the central nervous system can integrate internal (e.g., glycemia, menstruation hormones) and external signals that can impact brain control of bone formation and remodeling [51]. For example, experimental denervation of sensory and sympathetic nerve fibers can impact bone development and remodeling [55,56]. While nerve fibers of sympathetic, parasympathetic, and somatic origin also innervate jaw-bones including extraction sockets and peri-implant tissues [53,57,58,59,60,61], no information is available on the role of the nervous system in the healing and remodeling of jaw bones.

Recent studies have also shown important functional links between the central nervous system and the immune system that as we have discussed above plays a key role in peri-implant bone healing. Immune organs, such as lymphoid organs (e.g., lymph nodes, spleen) are innervated by sympathetic and parasympathetic nerve fibers of the autonomic nervous system which can in turn control bone remodeling [52,62]. The notion that the brain can modulate the immune response is also supported by studies showing the effects of mental and physical stress on the general health and immunity [63]. Moreover, usage of central nervous system medications (e.g., opioids, antidepressants, anticonvulsants) as well as depression conditions are associated with low bone mass and increased risk of osteoporosis and fractures [64]. It is interesting to note that recent studies have shown that impaired osseointegration and failures of dental implants are higher in patients treated with antidepressant drugs (selective serotonin reuptake inhibitors) [65,66,67]. However, it is unclear if the increased loss of osseointegration is produced by the drug or by the mental health condition itself and the impaired communication between the nervous system, immune system and bone healing and remodeling and thus, more robust research is required to identify the exact cause of osseointegration loss.

Another evidence for the role of the brain in bone growth in general comes from the effects of growth hormones in regulating bones growth during development, and bone remodeling throughout life. Growth hormones are secreted from the cerebral pituitary gland under the control of the cerebral hypothalamus. In fact, growth hormones can induce proliferation and activation of both osteoblasts and osteoclasts with an overall net effect of either bone growth, bone resorption or homeostasis [68]. Furthermore, growth hormones play a crucial role in fracture healing, and novel therapeutic approaches utilizing growth hormones (and other growth factors) to improve long bone healing are currently under investigation and development [69]. 

Altogether, the clinical significance of these studies on brain–bone axis (Figure 2) lies in them providing novel potential therapeutic targets for modulating bone remodeling. Thus, research is needed to gain a better understanding of the possible role of brain–immune system interaction also on jaw-bone remodeling and peri-implant bone healing and CBL.

## 3. Section B. Methods of Measuring Crestal Bone Loss

### 3.1. Current Methods

The need for measuring CBL has come with the spectrum of osseoseparation and peri-implantitis. A “lifetime” treatment for a patient requires osseosufficiency, i.e., the harmonious relationship between the host, the implant, and the clinician [3]. The rise of osseointegration science (and related expectations) led to the preservation of crestal bone height after implant placement in the context of quantitative success criteria for implant osseointegration. Monitoring changes in the bony anchorage routinely, at regular intervals, was advocated [1]. In this context, X-ray imaging techniques naturally emerged as a convenient tool for characterizing the marginal bone loss.

Change in bone height (loss or gain) represents the difference in bone levels at the same site at separate time-points. The initial reference bone level value is subtracted from each of the later values, usually but not always, resulting in a negative measurement representing loss of crestal bone. The initial reference is often recorded either right after implant placement (post-operative value) or once the implant becomes functionally loaded (prosthetic loading). These calculations compare the vertical distance between the crestal bone level at the implant contact and a reference point on the implant (implant platform for example), and as a consequence, should be referred as “distance to bone” values than to “bone level” values. Clinical routine measurements are often reported at the tenth of millimeter, while experimental ones may exhibit more accuracy.

To be consistent, the use of a single technique for both measurements is recommended (same imaging materials, same settings and same measure method). Then the known implant diameter, platform diameter, implant length or distance between two threads of screw-type implants may be used for calibration [70].

In the scientific literature, various imaging techniques have been used for measuring CBL, such as standardized intraoral radiographs (SIR), panoramic radiographs, computerized tomography scans, and cone beam computerized tomography (CBCT) scans [71]. The accuracy of the measurements have usually been assessed on jaws from animals or human cadavers, and those studies have repeatedly showed that panoramic radiographs lack reproducibility and resolution due to structure distortions and superimpositions, while computed tomography scans are affected by metal artifacts combined with an excessive exposure dose [71]. Today, SIR and CBCT appear to be the appropriate methods for routine assessment of crestal bone levels on living patients.

#### 3.1.1. Standardized Intraoral Radiographs (SIR)

Standardized intraoral (or periapical) radiographs have historically been, and remain to be, the most commonly used method for longitudinal assessment of peri-implant bone loss. For limiting distortion, the long cone paralleling technique is preferred to the intra-oral bisecting angle technique [72,73]. This technique, routinely used in periodontology, consists in holding the radiographic film parallel to the long axis of the implant and placing the X-ray beam perpendicularly to the receptor [74]. This paralleling technique requires the use of a film holder for routine clinical care, but for research purpose, a customized occlusal bite jig may be also fabricated to standardize the procurement of the implant image at different time points. The bite jig improves comparative measurements by limiting the parallax effect (apparent displacement of bony structures when radiographs are taken from different angles). The bite jig, typically fabricated from silicone, wax, or resin, is a repositioning key that fits to the film holder and can be stored by the dentist until next use. In some clinical studies, the bite jig is clipped on the attachment (locator, ball) or screwed into the implant. However, bone level interventions are not advocated as they may predispose to the bone loss. Some interesting devices have been described for the assessment of functional implants, such as a bite jig designed to be perpendicular to the initial implant placement driver [75].

Periapical radiographs used to be obtained on conventional films; however, the use of digital radiography is expanding in dental practice. When routine measurements are performed on conventional films, a magnifying lens can be used. Nowadays however, most research protocols incorporate high-resolution digitalization of a conventionally-obtained radiograph film. When routine measurements are performed directly with digital radiography, a sliding gauge tool can be used with most of the currently-available radiograph-related software to assess the distance between the crestal bone and the implant reference-point chosen. For research purposes, a method called the digital subtraction technique has been developed to directly measure bone loss by superimposing two serial radiographic images before subtracting them to isolate/quantify bone changes using specially-designed software [72].

##### Accuracy

Measurement accuracy is the closeness of agreement between measured and the true bone level. The accuracy relies not only on the resolution and sharpness of the radiographic material, but also on many clinical parameters, such as the degree of CBL, the jaw anatomy and configuration, the delay between placement and function, and the quantity of serial radiographs on the same implant [76].

When using a magnifying lens, e.g. ×10, with conventional SIR, inter- and intra-observer variability were shown to be approximately 0.14 mm and 0.08 mm respectively [76]. Conventional film and digital radiography exhibit the same accuracy [77]. Digitized conventional films may exhibit more noise artifacts and may lose density range but still provide comparable measurements [77,78].

##### Sensitivity and Specificity

In our context, the sensitivity of a radiographic technique consists in detecting the presence of crestal bone, while its specificity is about correctly detecting the bone (or defect) absence. These parameters have been tested in animals or in cadaver studies, when bone level estimations can be compared to the physical measurements. In a recent meta-analysis pooling the results of 5 studies, the SIR exhibited clinically acceptable sensitivity (60% when pooled; 56–100%) and specificity (59% when pooled; 51–98%) [71]. SIR detected more precisely large defects (around 3 mm) than small ones (1–2 mm) [71,79,80]. As a consequence, many authors reported the proximal bone loss to be underestimated by SIR measurements [81,82,83].

##### Pros and Cons

The primary advantages of SIR are the low exposure dose and being the least invasive of all the radiographic techniques. Combined with its low cost, the reliability of linear distance measurements, easy access and easy handling for dentists, this technique remains the gold standard for routine clinical measurements. 

However, only the mesial and distal CBL can be assessed with this technique. Furthermore, in the context of peri-implantitis, proximal bone levels were often shown to be more apical than the radiographically measured ones [81,82,83]. The tangential measurements can be affected by geometric distortions and anatomical superimpositions, especially since a strict parallel projection is difficult to obtain in some clinical situations [84]. In addition, SIR do not allow identification of the 3D morphology of a bone defect (intra-bony and supracrestal components) that influences diagnosis, prognosis and treatment planning [83,85,86].

#### 3.1.2. Cone Beam Computerized Tomography (CBCT)

The use of CBCT, also called digital volume tomography, to assess peri-implant bone level is more recent as this technology emerged in dentistry only 20 years ago. Compared with traditional CT, the lower irradiation dose and less severe metallic artifacts raised opportunities for new dental applications. 

In comparison with SIRs, CBCT image quality relies mainly on the technological performance of the material. Some of the most influencing parameters are the voxel size and the field of view. Indeed, image resolution is related to the size of volume elements, called voxels, which are often cubes (with edge ranging from 0.08–0.3 mm in research studies on peri-implant defects). However, small voxels come with additional noise [87]. Also, the field of view defines the volume of interest undergoing examination (cube ranging from 4 × 4 to 8 × 8 cm) and influences accuracy. This technological parameter is determined by the available detector, beam projection geometry and beam collimation. Small voxels and small fields of view improve measurements; but seeking the most precise peri-implant morphology when combining these two parameters will still deliver high radiation levels [80,88]. Image reconstruction parameters and filter software (used to lower metal artifacts) also influence the performance quality of the peri-implant measurements [89,90].

##### Accuracy

As previously mentioned, CBCT accuracy is defined by the field of view size, but also by the device scan mode and arc of rotation [91,92,93]. Indeed, the full-scan mode (360°) provides a higher diagnostic accuracy for peri-implant defects [90]. A recent systematic review concluded that large defects are more accurately detected than small ones, and that circumferential and fenestration peri-implant defects are more accurately detected than dehiscence defects [94].

Experimental measurements of peri-implant defects showed very low deviation when compared with direct measurement (0.18 ± 0.12 mm) and the proximal values were comparable to those obtained with periapical radiographs [95,96]. The spatial resolution can reach around 150–200 µm [72,97].

##### Sensitivity and Specificity

In a recent meta-analysis pooling the results of 9 studies, the CBCT exhibited clinically acceptable sensitivity (59% when pooled; 28–97%) and specificity (67% when pooled; 25–97%) [71]. Sensitivity globally increases with small voxels but remains challenged by small defects [90]. On the other hand, some authors suggested that specificity may increase with bigger voxels [80]. Filters can improve the detection of true positive or negative values [90].

##### Pros & Cons

When compared with SIR, CBCT delivers more radiation to patients, is more expensive for the patient and for the medical team, and has relatively limited availability. Metal artifacts (streaking, beam hardening, or scatter) increase with CBCT low energy settings and may add some false-positive bone on the vestibular side and false-negative bone on the other sides [96].

Both SIR and CBCT are interesting and validated imaging techniques for measuring peri-implant CBL. Their accuracy, sensitivity, and specificity are clinically acceptable [71,98]. On one hand, SIR provides only proximal values, but the data obtained often are sufficient to confirm changes in peri-implant CBL. On the other hand, CBCT exposes the patient to higher cost and radiation dose but offers a 3D characterization of the peri-implant defect. For these reasons, SIR remains the gold standard for routine assessment of bone level changes and for helping in peri-implantitis diagnosis, while CBCT is still confined to providing clear 3D images of diagnosed peri-implantitis that require a treatment plan [71,94,99]. In the future, CBL assessment may not be in X-ray imaging but rather in non-invasive 3D procedures such as ultrasound [100].

### 3.2. Novel Method: Photoacoustic Ultrasound as an Innovative Method to Measure Peri-Implant Pocket Depths and Bone Loss over Time

Ultrasound is the most widely used clinical imaging modality in medicine but has limited deployment in dental and periodontal practices [101]. In recent years, however, the number of preclinical dental applications of ultrasound has been increasing [102]. The advantages of ultrasound include the ability to image soft tissues in real-time without ionizing radiation at a relatively low cost. 

**Figure 3:**
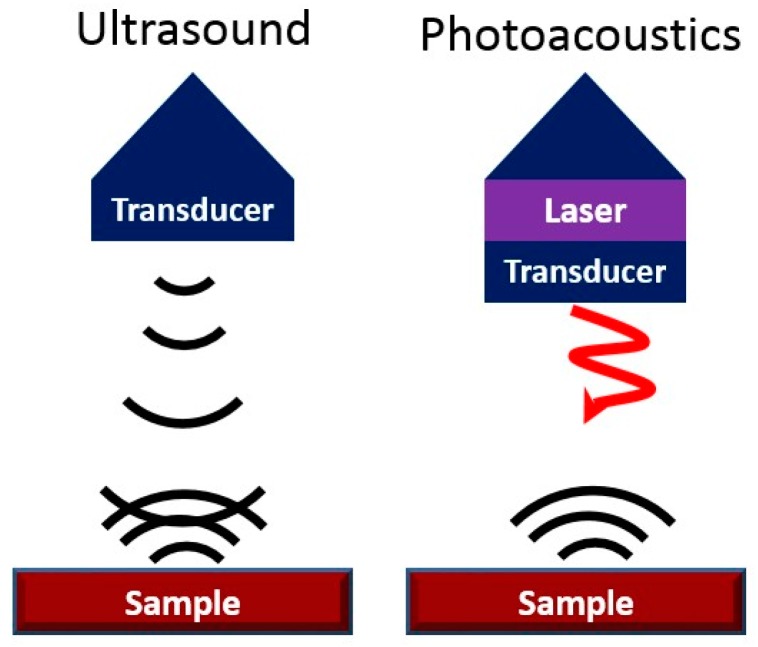
Acoustic Modalities. Ultrasound uses echoes to create contrast (“sound in/sound out”). Photoacoustics is “light in/sound out” and is based on thermal expansion of the target tissue or contrast agent.

One of the drawbacks of ultrasound is its limited contrast (signal from target versus signal from background). Contrast in conventional ultrasound is a function in differences in the acoustic impedance of different tissue types. Photoacoustic imaging is a hybrid form of ultrasound that can overcome this limitation and increase the contrast of ultrasound (Figure 3) [103]. It has a rapidly growing number of applications and uses optical—rather than acoustic—excitation to harness the photoacoustic effect. Photoacoustic imaging converts the incident light into sound following absorption and thermoelastic expansion of a target material [104]. It combines the good spatial and temporal resolution of ultrasound with the contrast and spectral imaging capabilities of optics. Typically, the optical excitation source (5–50 ns pulses at ~ 5 Hz) is a pulsed near-infrared laser (Nd:YAG/OPO) but low-power LED sources can also be used [105]. These pulses are absorbed by tissue, and the energy is released acoustically and detected by ultrasound transducers with center frequencies in the MHz range. The coupling of fiber optics with ultrasound transducers allows simultaneous ultrasound and photoacoustic imaging [106]. A variety of algorithms can be used for image reconstruction to maximize contrast, resolution, and signal-to-noise [107,108,109,110]. 

The most common uses of photoacoustic imaging are image-guided therapies [111], diagnosis of disease states [112,113], surgeries [114,115], and drug delivery [116,117]. These applications can be achieved through either endogenous or exogenous contrast. Endogenous contrast is based on the optical absorption of naturally occurring targets such as oxygenated/deoxygenated hemoglobin, melanin, lipids, and water [118]. Exogenous contrast mechanisms leverage the absorption of materials such as small-molecule dyes, fluorophores, and nanoparticles that originate from outside of the body [119]. In both cases, because photoacoustic intensity is proportional to optical absorption, light sources with specific wavelengths can be used for spectral differentiation between materials according to their absorption spectra. 

#### Imaging the Periodontal Pocket with Photoacoustic Ultrasound

Assessment of periodontal disease uses physical measurements (e.g., attachment level, probing depth, bone loss, mobility, recession, and degree of inflammation) [120]. Periodontal probing offers a numerical metric that reflects the extent of apical epithelial attachment relative to the gingival margin [121] but suffers from poor reproducibility due to variation in probing force [122]. Indeed, a recent meta-analysis showed a wide range of probing forces (51 to 995 N/cm^2^)—a variation of ~20-fold [123]. Other error sources include variation in the insertion point, probe angulation, the patient’s overall gingival health (weakly inflamed tissue), and the presence of calculus [121,124]. Thus, the exam is subject to large errors with inter-operator variation as high as 40% with r values between technicians <0.80 [125]. These errors can hamper clinical decision-making and epidemiological studies ultimately resulting in poor patient outcomes [126]. Furthermore, many patients find probing to be uncomfortable or painful—this can prevent patients from seeking care [127,128]. Moreover, the periodontal probing is time consuming for the practitioners. It is perhaps not surprising that periodontal examination was not performed in 50–90% of the audited dental records offices [129,130,131]. Finally, the benefit of traditional periodontal probing around implants is abrogated due to implant threads that impede probe penetration along the implant surface [132,133]. This limits the clinical assessment of these tissues, potentially leading to peri-implantitis [134,135]. 

The first study to use photoacoustic imaging for visualizing pocket depths was conducted by Lin et al. in 2017 [136]. A commercially available tomographic system (Visualsonics Vevo LAZR) was used for imaging porcine jaws extracted from frozen cadavers. A food-grade contrast agent containing melanin nanoparticles derived from cuttlefish ink was used to increase the photoacoustic signal of the pockets. This material acted as a safe, highly absorbing material capable of filling the gingival sulcus following oral irrigation. It had broad absorbance and photoacoustic signal.

**Figure 4:**
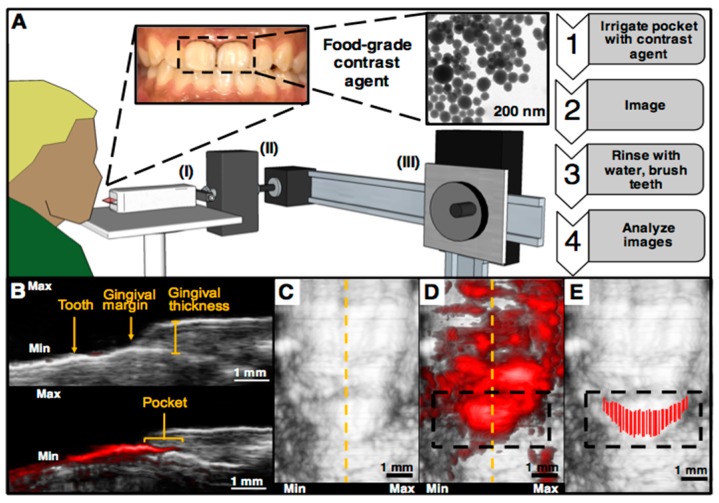
Representative human data of photoacoustic-ultrasound imaging for pocket depth measurements. (**A**) Overview of the imaging setup and methodology. The subject was seated in front of the transducer (I) and ultrasound gel was used for coupling. The stepper motor (II) was used for scanning the transducer and the sliding frame allowed positioning (III). First, the teeth of interest were irrigated with the contrast agent followed by imaging, removal of the agent, and image analysis. (**B**) A sagittal cross-section (dashed yellow line in Panel C) of a mandibular central incisor before (top) and after (before) irrigation with the contrast agent, revealing the pocket depth, measured from the gingival margin to the edge of photoacoustic signal. Nonspecific signal from the tooth, caused by the movement of coupling gel during scanning, did not contribute to the measurement. (**C**) A frontal view of the same tooth before (**D**) and after (**E**) irrigation. Nonspecific signal from contrast agent was removed during image processing by measuring the pocket from each sagittal plane as in Panel B and overlaying each measurement on the ultrasound-only image.

This technique was recently expanded to a healthy young adult case [137]. The same imaging system was adapted so that a subject could be scanned while seated (Figure 4A). Here, ultrasound gel was used for coupling and a medical head immobilizer and cheek retractors were used to minimize movements from the subject; 40 MHz ultrasound was used throughout. Again, the procedure began with irrigation of the pocket followed by laser pulsing and imaging, removal of the contrast agent, and image processing. The pocket depth could be visualized for a given sagittal plane (Figure 4B–D) after administration of the agent. Because these experiments used ultrasound gel for coupling, it was common during scanning for the agent to nonspecifically coat the surface of tooth. However, this nonspecific signal could be removed in post-processing by using the ultrasound-only images to locate the gingival margin. Any signal originating from tooth surface occlusal to the margin was ignored allowing a final mapping of the pocket to be manually generated (Figure 4E). In the future, this processing step will be automated. 

In the case of implants, physical probing is typically hindered by threads that impede probe penetration along the implant surface [132,133]. This limits the ability for clinical assessment of these tissues potentially leading to peri-implantitis [134,135]. We note that photoacoustic ultrasound has not been explicitly tested yet for imaging the pockets around implants. However, because it relies on the flow of contrast agent into the pocket rather than the physical penetration of a metal probe, the presence of implant threads should not affect measurements. For this reason, we believe photoacoustic imaging is promising for patients with implants that obstruct manual probing. Of course, additional work remains to improve the clinical feasibility of the technique including development of a mouthpiece transducer and the implementation of more affordable and stable excitation sources, such as LEDs or laser diodes.

## 4. Summary

New ways of appreciating CBL are blending traditional etiologies with novel mechanisms that better reconcile what was originally thought to be taking place during osseointegration with actual long-term clinical outcomes. Today, the ability to look back on osseointegration outcomes at the implant level, the prosthesis level, the patient level and even the clinician level allow us to recognize that osseointegration likely represents a form of foreign body reaction and focuses our attention on elements that, therefore, influence the immune response or the consequence of a patient’s immune response. In this way, traditional etiologies such as inflammation from infection and overloading can be viewed as modulators of the immune response and the effect of immune response through neuroimmunomodulation opens up new and exciting avenues for future research.

Clinically, measuring crystal bone loss remains at the mercy of the constraints of radiographic imaging. Nevertheless, new methodologies and digital technologies portend the introduction of non-invasive methods that may be more sensitive and specific with regard to measurement of crestal bone position and changes in crestal bone position over time. Here too, innovations in imaging will allow us to better assess the effect of new techniques, products, protocols and materials.

## Figures and Tables

**Table 1:** Animal experiments about biological complications related to implant loading.

Year	Animal Model	Loading Pattern	Bone Resorption	Healing Period	Loading Period	Implant System
Isidor [9]	Monkey mandible	10–300 N330 N/s for 5 days	Yes	6 months	4–15 months	Astra
Miyata et al. [10]	Monkey mandible	Supra-occlusal contact	Yes	3.5 months	4 weeks	Intra-mobile element (IMZ)
Heitz-Mayfield et al. [12]	Dog mandible	Supra-occlusal contact	No	6 months	8 months	Straumann
Esaki et al. [11]	Dog mandible	Immediate load	Yes	None	3 weeks	Branemark
